# Vascular injuries after bear attacks: Incidence, surgical challenges and outcome

**DOI:** 10.4103/0974-2700.76827

**Published:** 2011

**Authors:** Mohd Lateef Wani, Abdul Gani Ahangar, Gh Nabi Lone, Reyaz Ahmad Lone, Hakeem Zubair Ashraf, Abdul Majeed Dar, M A Bhat, Shyam Singh, Akram Hussain Bijli, Ifat Irshad

**Affiliations:** Department of Cardiovascular and Thoracic Surgery, SKIMS, Soura, Kashmir – 190 011, India; 1 Department of Plastic Surgery, SKIMS, Soura, Kashmir – 190 011, India; 2 Department of Radiodiagnosis, SKIMS, Soura, Kashmir – 190 011, India

**Keywords:** Bear maul, saphenous vein, vascular injury

## Abstract

**Background:**

Bear mauling is rarely reported in medical literature due to its rare occurrence. Present study was undertaken to describe the pattern and management of bear maul vascular injuries in Kashmir.

**Patients and Methods:**

Study of patients with bear maul vascular injury from 1^st^ Jan 2004 to 31^st^ Dec. 2008. Fifteen patients with bear maul vascular injury were studied. All patients of bear maul without vascular injury were excluded from the study.

**Results:**

Most of the patients were treated by reverse saphenous vein graft or end to end anastomosis. Most common complication was wound infection (20%) followed by graft occlusion (13.33%). There was no operative death.

**Conclusion:**

Bear attacks are very common in Kashmir. Vascular injury due to bear maul needs prompt resuscitation and revascularization. Results are very good provided timely intervention for revascularization is done.

## INTRODUCTION

Kashmir valley is surrounded by dense forest range, a habitat for lot of wild animals especially Bear, Leopard, Monkeys etc. Lot of deforestation has taken place in past two decades as a result of law and order problem in the valley. Since bear is the commonest inhabitant of these forests, lots of them come down to densely populated area in the neighborhood of these forests resulting in lot of bear mauling.

Black bear is more common in Kashmir as compared to Grizzly Bear. Black bear generally weigh 55 to 135 kg and are usually 1.2 to 1.5 m long. They easily reach 1.8 m height when standing on their hind legs and readily climb trees and sprint at speeds of up to 48 km/h on flat terrain.[1]

Studies on human injury as a result of bear attack are few, and vascular injury by such attacks is seldom reported. Bear mauling has taken an epidemic proportion in Kashmir especially in the months of July, August, and September. Areas near forest range are commonly affected by bear mauling. Since the conflict usually occur in remote areas that involve a substantial delay with respect to notification, rescue and definitive care. Plastic surgery department of our hospital usually take care of these injuries except in case of head injury and vascular injury where neurosurgery and cardiovascular surgery are called in respectively. Our study presents an analysis of bear attacks having vascular injury. Objective was to study the pattern, presentation and management of bear maul vascular injuries.

## PATIENTS AND METHODS

The study was done in the Department of Cardiovascular and Thoracic Surgery Sher-i-Kashmir Institute of Medical Sciences Srinagar. All patients with bear maul vascular injuries were studied between 1^st^ Jan 2004 and 31 Dec 2007.

All patients were initially resuscitated in emergency reception. Analgesia and tetanus toxoid injections were administered to every victim. Apart from systemic examination for associated injuries, initial limb evaluation included color, temperature, capillary refilling, and pulse.

There were 212 cases of bear maul during this period. Out of them 15 cases (7.07%) had vascular injury. All these patients had unprovoked bear attacks. All these injuries were by clawing only. Vessels below ulnar and radial artery in upper limb and tibial arteries in lower limb were excluded from the study. All patients of bear maul without vascular injury were excluded from the study.

Of the 15 patients three patients were in shock. They had systolic blood pressure of less than 80 mmHg. Rest of the patients were hemodynamically stable. However all 15 patients had tachycardia on presentation.

All our patients presented with hard signs of vascular injury. Vascular injury in most of the cases was obvious. Two patients with expanding neck hematoma and one with absent distal pulse were subjected to Color Doppler ultrasound before exploration. All patients were operated in emergency. All patients received ceftriaxone sulbactam and tinidazole at induction and exploration was carried under general anesthesia. Proximal and distal control of vessel was achieved using standard operative approaches. The injured vessel was isolated and exact extent of damage was established. Thorough debridement of soft tissue and copious saline irrigation was done before repair. Injury with <2 cm loss of vessel wall was managed by end to end anastomosis. Injury with >2 cm loss of vessel wall was managed by reverse saphenous venous graft from contralateral limb. A Fogarty catheter was used to retrieve thrombus in case of absence of adequate flow. Venous repair was done by lateral repair if associated. Associated median nerve was repaired in one case by plastic surgeons. Adequate soft tissue coverage was ensured. No standard prophylactic fasciotomy was done in any patient. However of all patients these patients had deep soft tissue lacerations around the site of vascular injury. Associated injuries were taken due care of. Table 1 shows associated injuries of the patients. All patients received Heparin 10 000 IU S/C 8 hourly in postoperative period besides antibiotics. All patients were subjected to Color Doppler study on the 10^th^ postoperative day to ensure the patency of the vessel. All of the patients received prophylaxis against Rabies virus. Mean hospital stay was 12.3 days. All patients were put on Aspirin for 3 to 4 weeks.

**Table 1:** Associated injury

Associated injury	Number of patients
Multiple laceration on face	8
Fracture nasal bones	2
Fracture of Zygoma	1
Laceration of scalp	5
Laceration in inguinal region	6
Fracture mandible	1
Miscellaneous injuries	4

## RESULTS

Median age was 36.4 years. Male patients accounted for 10 cases (66%). All patients presented within 4 h of injury. Most patients (9 cases) were referred with visible pulsatile arterial bleed from peripheral hospitals, three patients presented with shock, two patients presented with expanding neck hematoma, one patient had absent distal pulse [Table 2]. Four patients had femoral artery injury, three patients had popliteal artery injury, six patients had brachial artery injury and two patients had common carotid artery injury [Table 2]. Three of the patients had associated venous injury. Among them two had popliteal vein injury and one had external jugular vein injury. One of the patients had associated median nerve injury. All of the patients had associated soft tissue injuries such as laceration on face, scalp, etc.

**Table 2:** Presentation, artery involved, and procedures performed

Presentation	No.	Artery involved	No.	Revascularization Procedure	No.
Arterial bleed	9	Femoral	4	End to end repair	3
Shock	3	Popliteal	3	Lateral repair	2
Expanding neck hematoma	2	Brachial	6	Reverse saphenous Vein graft	10
Absent peripheral pulse	1	Carotid	2	Lateral Venorrhaphy	3

Most of the patients were treated with sphenoid venous graft or end to end anastomosis Table 2. Wound infection was the most common complication that occurred in three patients (20%), followed by graft occlusion in two patients (13%). Wound infection was treated by daily dressings and change of antibiotic as per swab culture sensitivity. Most of the cultures had grown pseudomonas due to sensitivity to ciprofloxacin that was administered. Graft occlusion was treated by thromboembolectomy successfully. They did well postoperatively. One of the patients who had a popliteal artery repair developed joint stiffness, which was successfully managed by physiotherapy. Patient who had a median nerve repair done had Grade IV muscle power with the recovery of protective sensation at one-year follow-up. None of the patients had compartment syndrome. Amputation rate was zero. There was no other complication. Associated injuries were taken care of. There were no operative deaths.

## DISCUSSION

Dog bitesare reported to constitute about 50-90% of all animal-inflicted injuries,[2–5] but in Kashmir, victims ofbear and leopard attacks constitute the majority ofthe patients requiring medical attention.[6] Present study was done to report the very unusual cause of vascular injury which shows that vascular injury due to bear attacks has an excellent outcome, provided prompt resuscitation and revascularization is done. Vascular injury due to such attacks is usually obvious and hence requires no preoperative angiography. To our best knowledge this is the first reported series of vascular injuries after bear attacks.

Most of vascular injuries are caused by penetrating injuries or road traffic accidents. Most of the data on vascular trauma is from major wars i.e. World War I, World War II, Korean conflict, Vietnam War, Gulf War I and II, besides low level civilian wars in Middle East, Yugoslavia, Russian Republic, Kashmir and Central Africa.

Murphy in 1896 did the first successful end to end vascular anastomosis in man.[7] The successful repair of vascular injuries in Korean conflict is a pleasant contrast to the experience of World War II, because of substantial progress in techniques of vascular repair accompanied by improvement in anesthesia, blood transfusion, and use of antibiotics.[8,9] Vascular injury due to bear attacks is seldom seen. Early recognition of these injuries is essential in the management. The classical features of vascular injury are usually obvious in these cases. Routine preoperative investigation is unnecessary. A cardinal operative principle in managing vascular trauma is to obtain proximal and distal control of injured vessel before entering surrounding hematoma.[10] In extremities as in neck, control is achieved using standard extensile vascular exposure techniques.[11,12] Once proximal and distal control of vessel was achieved, irrigation of distal arterial tree with heparinized saline (25–50 IU/ml) to remove or dislodge small thrombi from the main arterial tree was done. Fogarty catheters were used as and when needed. Repair of injured vessel by primary end to end anastomosis after freshening of the edges was done in cases with no or minimal segmental loss (<2 cm loss). Reverse saphenous vein graft from contralateral limb was used in case of segmental loss of >2 cm. Small lacerations were treated by primary repair. Systemic anticoagulation in the form of subcutaneous heparin was administered soon after surgery and continued postoperatively for one week. It was followed by oral aspirin for 3 to 4 weeks. Popliteal vein repair was done, as we like many others[13,14] advise repair of popliteal vein so that its repair will enhance the success of arterial reconstruction. However popliteal vein has been successfully ligated by some authors with no complications.[15,16] However arterial repair preceded the venous repair to decrease ischemia time.

As reported by many authors,[14,17–23] the significant factor associated with increased limb loss is the time lapse between injury and operation. They say with delay there is progression of muscle ischemia, small vessel thrombosis that prevents successful outcome of repair. In our study, all patients presented to hospital within four hours of injury and were revascularized within 8 h of injury. Our limb salvage was 100% as none of our patients had warm ischemia time of more than 8 h.

Another important factor contributing to limb loss is associated fracture.[20,24] However none of our patients had associated fracture.

## CONCLUSION

1. Bear attacks has increased in Kashmir Valley resulting in large number of injuries ranging from simple laceration to vascular injury.
2. Vascular injury by bear attack has a very good outcome probably because of absence of cavitational effect, provided prompt resuscitation, revascularization and proper technique of vascular repair is followed.

## References

[1] Kunelius R (1983). Animals of the Rockies. Banff. Alberta: Altitude.

[2] Freer L (2004). North American wild mammalian injuries. Emerg Med Clin North Am.

[3] Bahram R, Burke JE, Lanzi GL (2004). Head and neck injury from a leopard attack: Case report and review of the literature. J Oral Maxillofac Surg.

[4] Wiens MB, Harrison PB (1996). Big cat attack: A case study. J Trauma.

[5] MacBean CE, Taylor DM, Ashby K (2007). Animal and human bite injuries in Victoria, 1998-2004. Med J Aust.

[6] Dhar SA, Butt MF, Farooq M, Mir MR, Wani ZA, Afzal S (2008). Pattern of orthopaedic injuries in bear attacks: Report from a tertiary care centre in Kashmir. Injury.

[7] Murphy JB (1897). Resection of arteries and veins injured in continuity: End-to-end suture, experimental and clinical research. Med Rec.

[8] Debakey ME, Simeone FA (1946). Battle Injuries of the Arteries in World War II: An analysis of 2,471 cases. Ann Surg.

[9] Hughes CW (1954). Acute vascular trauma in Korean War casualties: An analysis of 180 cases. Surg Gynecol Obstet.

[10] Rich NM (1970). Vascular trauma in Vietnam. J Cardio Vasc Surg.

[11] Hirshberg A, Wall MJ, Johnston RH, Burch JM, Mattox KL (1994). Transcervical gunshot injuries. Am J Surg.

[12] Valentine RJ, Wind GG (2003). Anatomical exposure in vascular surgery.

[13] Sullivam WG, Thornton FH, Baker LH, Cohen A (1991). Early influence of popliteal vein repair in treatment of popliteal vessel injuries. Am J Surg.

[14] Daughterty ME, Sachatello CR, Ernst CB (1978). Improved treatment of arterial popliteal injuries. Arch Surg.

[15] Timberlake GA, O, Connell RC, Kerstein MD (1986). Venous injury: To repair or to ligate, the delimma. J Vasc Surg.

[16] Swetman JA, Hardin WD, Kerstein MD (1986). Successful management of bifercation injuries. Am J Surg.

[17] Orcutt MB, Levine BA, Root HD, Sirinek KR (1983). The continuing challenge of popliteal vascular injuries. Am J Surg.

[18] Dajani OM, Haddad FF, Hajj HA, Sfeir RE, Khoury GS (1988). Injuryto femoral vessels: The LebaneseWar experience. Eur J Vasc Surg.

[19] Dar AM, Ahanger AG, Wani RA, Bhat MA, Lone GN, Shah SH (2003). Popliteal artery injury: Kashmir Experience. J Trauma.

[20] McNamara JJ, Brief DK, Stremple JF, Wright JK (1973). Management of fractures with associated arterial injuries in combat casualities. J Trauma.

[21] Conkle DM, Richle RE, Sawyers JL, Scott HW (1975). Surgical treatment of popliteal artery injuries. Am J Surg.

[22] Menzolan JO, Logaerjo FW, Doyle JE (1982). Management of vascular injuries of leg. Am J Surg.

[23] Downs AR, McDonald P (1986). Popliteal artery injuries: Civilian experience with sixty- three patients during a twenty- four year period (1960-1984). J Vasc Surg.

[24] Odland MD, Gisbert VL, Gustilo RB, Neyo AL, Blake DP, Bubrick MP (1999). Combined orthopaedic and vascular surgery injury in the lower extremities: Indications for amputation. Surgery.

